# The puzzle of COVID-19 in Central America and Panama

**DOI:** 10.7189/jogh.11.03077

**Published:** 2021-06-05

**Authors:** Andy A Pearson, Andrea M Prado, Forrest D Colburn

**Affiliations:** 1 INCAE Business School, Alajuela, Costa Rica; 2 City University of New York, New York, New York, USA

The novel coronavirus, SARS-CoV-2, which causes the infectious disease COVID-19, was declared by the World Health Organization (WHO) in March 2020 to be a pandemic. It is alarming how quickly and destructively it spread throughout the world. However, the disease has struck countries, and even regions, with varying intensity, raising questions about what makes populations vulnerable and about public policies' effectiveness to control the virus. The Central American isthmus embraces the five Central American countries (which were once a single political unit) and Panama (which was formerly a province of Colombia). These six countries are not only tucked together into a corner of the world, but they are all small countries sharing a relatively common history and culture. Nonetheless, the official data as of April 2021 on COVID-19 from each country in the region reveals that the six coutries have suffered unequally from the pandemic.

According to the official data, the countries with the highest economic development – Costa Rica and Panama – have fared the worst in terms of the number of cases and deaths per million inhabitants, not so in the mortality rate (percentage of cases resulting in death). Nicaragua, the second poorest country in the Western Hemisphere, has instead fared the best in terms of the number of cases and deaths per million inhabitants, not so in mortality rate. Official COVID-19 data in the region is likely to be unreliable and misleading – in some countries more than others. In particular, there are widespread doubts in the northern countries of the region about the reliability of COVID-19 data, hindering efforts to assess the magnitude of the challenge, to make public policy decisions, and to compare outcomes among countries.

Variables that might influence the scarcity and shortcomings of the data are numerous [1]. Perhaps wealthier countries with better health systems are more likely to notice (through testing) and record infections and deaths. At the same time, these countries might be better positioned to treat COVID-19 patients and achieve lower case mortality rates than those with weaker health systems. Perhaps governments, particularly in prosperous democracies, are more accountable to citizens and offer more complete and reliable information. Despite these shortcomings in some countries' official data, it is helpful to discuss them and share health care professionals' views to complement these statistics. In addition, it is useful to assess the potential unreliability of COVID-19 data about fatalities by measuring the magnitude of the underestimation of deaths in official COVID-19 data.

Given that the pandemic is so pervasive, it is instructive to explore the political, economic, and social conditions that shape the region’s vulnerability to COVID-19. Politics, urban density, and the level – and kind – of economic development shape each countries’ exposure to the virus and ability to mitigate its reach. For example, democracies might be more vulnerable because citizens exercise their liberties, including continually moving in and out of many different social and economic circles. Conversely, authoritarian regimes might find it easier to restrict, abruptly, social movement and economic activity. However, such regimes might also be tempted to manipulate data for political expediency. Reaching hard-fast conclusions about these kinds of variables in the Central American isthmus is difficult, but such discussions are necessary. The views of health care practitioners are illuminating and can, perhaps, serve as a guide for further research.

**Figure:**
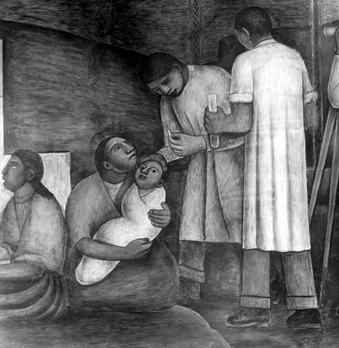
Photo: Photograph by Tina Modotti, view of a Mexican mural, showing a mother and child being helped by doctors. Circa 1928. Courtesy of Throckmorton Fine Art, New York.

Throughout the world there are challenges in understanding national vulnerabilities to COVID-19. Indeed, there is, worldwide, a growing appreciation for what has been named “the COVID-19 conundrum: Why does the pandemic seem far deadlier in some countries than in others?” [2]. Answering this all-important question requires good and extensive data to generate and test hypotheses. Unfortunately, for many countries, this data does not yet exist.

International organizations, such as the WHO or the World Bank, have yet to study systematically COVID-19 in the region. Surprisingly, even the media in this isthmus has given scant coverage to disparities in COVID-19 infection rates among neighboring countries. Interviews with health professionals in each of the six countries are illuminating, revealing that those in the medical profession are aware of what their colleagues in the region are confronting. These interviews sought to be a complement to the statistical data and provide insights into what professionals in the field perceive or believe is happening. Those interviewed included doctors at the “frontline” of hospitals and clinics, health professionals working at the community level in non-governmental organizations, and current or former public officials from the Ministries of Health or the Social Security Administration. Those interviewed were cautious in reaching conclusions. Time and time again, it was stated that much was not only unknown but that it would always remain unknown. Even the number of cases and deaths from the disease were deemed unclear; data are not judged reliable, especially in Nicaragua, El Salvador, Honduras, and Guatemala (four of the six cases).

The six countries of the Central American isthmus have long been placed in three groups. One group consists of Costa Rica and Panama, the two southern countries that have by far the highest per capita incomes, stable democracies, and decades of continuous investment in social services, notably education and health care. The second group consists of only Nicaragua, a particular case. The country was torn apart by a revolution in 1979 that led to a collapse in per capita income (the country is now the poorest country in the Americas after Haiti). Nicaragua also continues to be marred by poor governance and political strife. The third group in the isthmus is the “Northern Triangle”: Guatemala, El Salvador, and Honduras. These three countries are poor and have never made sustained investments in social services to combat the poverty that confronts a large share of their inhabitants.

## DATA

**Table 1** shows the incidence of COVID-19 in the five Central American countries and Panama. The data are from the governments of each country, with the exception of Nicaragua. Official data from Nicaragua is judged completely unreliable [6]. As one Nicaraguan doctor quipped, “*The data the government is publishing are not fooling anyone*” (personal communication, October 22, 2020). In response, a group of doctors and citizens organized the COVID-19 Citizen Observatory in Nicaragua to report weekly data on infections and deaths believed to be caused by COVID-19. Somewhat surprisingly, the Nicaraguan government has not suppressed the group's activity, perhaps because its higher figures nonetheless suggest that Nicaragua has suffered the least in the isthmus from COVID-19. That outcome is notable and counterintuitive given the country's poverty and the government's dismissal of the epidemic (to the point of asking doctors and nurses not to wear masks because they scare the population) [7].

**Table 1:** COVID-19 cases, deaths, and tests reported for Central America to April 2021*

Country	Cases	COVID-19 # deaths	Tests	Case fatality rate	Cases/ 1M pop	Deaths/ 1M pop	Tests/ 1M pop	Population (in millions)
Guatemala	216 329	7309	1 187 042	3.4%	13 029	440	71 491	16.6
El Salvador	68 007	2089	894 988	3.1%	10 538	324	138 681	6.5
Honduras	203 359	4981	567 813	2.4%	20 866	511	58 260	9.7
Nicaragua	13 806	3055	–	22.1%	2109	467	–	6.5
Costa Rica	233 498	3115	919 324	1.3%	46 260	617	182 132	5.0
Panama	361 678	6196	2 321 435	1.7%	85 172	1 459	546 678	4.2

*Sources: Johns Hopkins Coronavirus Resource Center [3], Worldometers [4], April 22, 2021. Except for Nicaragua: Nicaraguan COVID-19 Citizen Observatory data [5].

What is most notable about the data presented in **Table 1** is that Costa Rica and Panama have suffered the most from COVID-19, both in the number of cases and of deaths per million inhabitants. It is also surprising that Honduras is the country in the Northern Triangle hardest hit by the pandemic. Health professionals in the region, when presented with the data, inevitably question its reliability. There are a number of concerns.

Wealthier countries, such as Costa Rica and Panama, are more likely to test for the disease—and to have testing available for the poor. And countries that are democratic (which is defined to include freedom of the press), such as, again, Costa Rica and Panama, are less likely to engage in manipulation of data for political purposes. Panama is praised for its Gorgas Institute for Health Studies and its decentralized capacity to perform tests throughout the country. A doctor in Panama likewise stresses that patients with positive test results are likely to receive multiple tests, a desired practice but one not followed elsewhere in the isthmus (personal communication, January 12, 2021). In Costa Rica, the Ministry of Health has a presence throughout the country, and it is trusted by Costa Ricans. Still, there are doubts even in Costa Rica and Panama of whether reporting of infections is complete [8]. Costa Rica has a large immigrant population of Nicaraguans (judged to be 10% of the population), and many do not have legal residency and so are hesitant to avail themselves of public health services. In Panama, there are isolated rural communities that may be beyond the reach of adequate health care services. Still, many health care professionals in the isthmus accept the conclusion of a Panamanian doctor who asserts, “*The data of Costa Rica and Panama are credible*” (personal communication, January 12, 2021).

It is in the poor northern countries of Nicaragua, Honduras, El Salvador, and Guatemala where there are widespread doubts about the reliability of data about COVID-19. In Nicaragua, a doctor reports, “*There are many people here suffering from the disease. But they do not seek medical attention; they remain anonymous*” (personal communication, October 22, 2020). Surprisingly, the doctor reports that he himself had COVID-19 but never took a test. In Honduras, a doctor reports that because of insufficient testing, “*We do not have an accurate diagnosis of the number of cases in Honduras*.” He adds, “*For those of us doctors who are attending cases day-after-day, we can see that official figures of COVID-19 cases should be double or triple what is reported*” (personal communication, October 27, 2020). In El Salvador, a doctor reports, “*The data that is reported is not correct: I do not know if it is being manipulated or not, but it is incorrect*” (personal communication, October 21, 2020). A colleague in Guatemala was blunt about the data for the most populous country in the isthmus: “*The data for Guatemala is not correct.*” He adds, “*The cases that are registered are only for the most serious cases, those requiring hospitalization*” (personal communication, October 23, 2020).

Explanations vary for a likely under-reporting of COVID-19 cases in the northern countries of the Central American isthmus. They include prominently insufficient testing. Public health services do not have the necessary resources for comprehensive testing. Private testing is expensive, reportedly as high as US$165.00 in Guatemala, a prohibitive cost for most Guatemalans. There is sometimes a fear of entering a hospital. More commonly, as a doctor in Honduras reports, “*People do not seek public health services because there are few doctors and little medicine*” (personal communication, October 27, 2020). Hence, as a Nicaraguan doctor says, “*People attend to themselves at home*” (personal communication, October 22, 2020). Sometimes, though, there is no alternative: in rural areas, where many Central Americans live, there is a paucity of health care facilities, public or private. Sometimes there is nowhere to seek assistance. Adding to difficulties, there is a widespread stigma to COVID-19, resulting in deaths being reported by family members as a “stroke” or “diabetics” when, in fact, it was from COVID-19.

By taking a country's total number of deaths from all causes reported for the year 2020 (a pandemic year) and substracting the average number of deaths in the last five normal years (2015-2019), one can get excess mortality, a more comprehensive measure of the total impact of the pandemic on deaths than the COVID-19 death count alone [9]. However, as the pandemic has discouraged people from seeking health care, including visiting hospitals, and also made it harder for doctors to treat all patients, some excess deaths might be attributed to diseases other than COVID-19. Nevertheless, if one assumes the excess number of deaths in 2020 are all attributable to COVID-19 and compares this number with the total COVID-19 deaths reported in 2020, one obtains an approximation of the maximum range of COVID-19 deaths that might have been under-estimated by country (**Table 2**).

**Table 2:** COVID-19 and non-COVID-19-related deaths in Central American countries, 2015-2020*

Country	Average # deaths per year (2015-2019)	Total # deaths (2020)	Excess # deaths (2020) vis-à-vis average 2015-2019	COVID-19 # deaths (2020)	% of excess deaths not reported as COVID-19
Guatemala	82 159	94 798	12 639	4813	61.9
El Salvador†	28 064	35 368	7304	717	90.2
Nicaragua†	15 706	23 581	7875	2565	67.4
Costa Rica	22 983	26 209	3226	2185	32.3
Panama	19 320	23 876	4556	3975	12.8

*Source: Giattino et al.[9], March 31, 2021.

†Excess deaths include data for January to August, 2020. For comparison purposes, COVID-19 deaths (2020) for both countries include the same time span.

Under these estimations, differences among countries' potential under-reporting are evident. Costa Rica and Panama seem to have the most reliable data in the region, while the northern countries have more than 60% of their excess deaths in 2020 not reported as COVID-19. El Salvador stands out for only reporting 10% of their excess deaths for 2020 as COVID-19, representing a potential 90% under-estimation of COVID-19 cases. Honduras does not have the data for total deaths for 2020, so it was impossible to include it in **Table 2**. Two more conditions might influence the under-reporting of COVID-19 deaths. First, among the poor, especially the rural poor, not all deaths are correctly registered as they might not have access to a health professional, and thus, it might not be possible to confirm the cause of death. Second, there is a final, and distinct suggested reason for the lack of reliable data: in the northern countries of the isthmus – Nicaragua, Honduras, El Salvador, and Guatemala – there is a conviction among health care professionals that political authorities manipulate data, with a strong tendency towards underreporting. No politician wants to be blamed for what might be portrayed as unnecessary illness and death.

Doubts about the reliability of COVID-19 data were the dominant concern of health care professionals interviewed. Those interviewed, though, also stress that need to understand the demographic, socio-economic conditions, and political realities that frame – and shape – the dynamics of COVID-19 in the region.

## NATIONAL VULNERABILITIES TO COVID-19

The first cases of COVID-19 emerged in Central America shortly after those registered in Asia and Europe. Local governments had little chance of strengthening their health systems or obtaining the necessary supplies to confront the crisis. **Table 3** offers an overview of each country's investment in health care, the availability of doctors, beds, and of the presence of diseases that are associated with severe cases of COVID-19, prominently diabetics, elevated blood pressure, and obesity. Costa Rica and Panama have higher percentages of the population over sixty-five years of age, and suffering from diabetics, high blood pressure, and obesity. On the other hand, their higher Gross National Products (GNP) translate into much higher expenditures for health care. (Costa Rica's per capita income, for example, is between six and eight times the per capita income of Nicaragua.) No one in the isthmus was prepared for the infectious disease, or even had an idea of how it would batter their country.

**Table 3:** Health care expenditures, resources, and prevalence of select diseases in Central America*

	Guatemala	El Salvador	Honduras	Nicaragua	Costa Rica	Panama
Current health expenditure (2017, % of GDP)	5.8	7.2	7.9	8.6	7.3	7.3
Current health expenditure per capita (2017, US$)	474.8	582.7	395.8	506.2	1,262.2	1,794.8
Medical doctors (2018, per 10 000 people)	3.5	15.7	3.1	9.8	28.9	15.7
Hospital beds (2015, per 1000 people)	0.6	1.3	0.7	0.9	1.2	2.3
Population 65 y of age and over (2019, % of total)	4.9	8.5	4.8	5.5	9.9	8.3
Prevalence of overweight adults BM I≥** **25 (2016, crude estimate, %)	51.4	56.8	51.9	54.8	61.5	58.5
Prevalence of raised blood pressure (SBP ≥ 140 OR DBP ≥** **90) (2015,18+ years, %)	17.0	16.8	17.3	17.2	18.2	19.3
Raised fasting blood glucose (≥** **7.0 mmol/L or on medication) (2014, crude estimate, %)	7.5	8.8	7.2	8.1	8.5	9.0

GDP – gross domestic product, BMI – body mass index, SBP – systolic blood pressure, DBP – diastolic blood pressure

*Sources: World Health Organization [10], World Bank [11].

The democracies of Costa Rica and Panama may have been more vulnerable to COVID-19 because their populations are accustomed to personal liberties and the opportunities to move freely about for any number of reasons. Governments would have found it difficult to implement draconian “lockdowns” (of the kind, for example, instituted in El Salvador) even if they had been so inclined. An accomplished doctor in Costa Rica said of Panama, “*Panamanians are absolutely irreverent; they thought the pandemic was a fable; they continued with their carnivals and parties. Panama is like the United States, and this relationship will never change. The United States is like the father that leads the way*” (personal communication, October 19, 2020). The doctor, though, might as well having been speaking of Costa Rica, where the openness and laxity of society is not far behind that of Panama.

A second vulnerability could come from the region's dense urban neighborhoods. All of the countries of the isthmus have a majority of their population living in urban settings. Costa Rica's population is five million, but well over two million Costa Ricans live in the greater metropolitan area of the capital, San José. Likewise, Panama City's metropolitan area is home to nearly half of Panama's four million people. Urban neighborhoods are often dense [12]. As a Costa Rican doctor explained, “*Everyone says to stay in your home, but no one notes that ‘home’ is ten by ten meters with eight people*” (personal communication, October 22, 2020).

The most densely populated country in the region is El Salvador. Two million, four hundred thousand Salvadorans live in the greater metropolitan area of the capital, San Salvador; the total population of the country is six million, two hundred thousand. Perhaps the density of El Salvador, and of San Salvador in particular, would have led to a higher COVID-19 infection rate, but the government of the country had an early and extended “lockdown,” which was vigorously enforced. (El Salvador is also believed by doctors to be the country in the region with the most government manipulation of data.)

In contrast, Guatemala, Honduras, and Nicaragua are decidedly more rural. And Nicaragua's capital, Managua, is widely spread out, with a paucity of office towers or apartment blocks. A Costa Rican doctor explained Nicaragua having the lowest incidence of COVID-19 infection in the isthmus by stating, “*It is a rural country, with a population very dispersed*” (personal communication, October 19, 2020).

A third proposition for explaining national vulnerability to COVID-19, level of economic development, is judged by health care professionals in the Central American isthmus to be complex. A relatively high level of economic development brings risks, such as with the business travel in Panama (some tied to the Panama Canal, some to banking or other business) and the international tourism of Costa Rica (the largest generator of foreign exchange for the country). Those who are prosperous in the isthmus are most likely to live in urban settings and to enjoy a wide variety of experiences, many of which can entail exposure to an infectious virus. Conversely, subsistence farming in the western highlands of Guatemala or in eastern Nicaragua may entail little risk. As a Guatemalan doctor put it, “*Isolated rural communities have been the least affected. There people do not travel; they work in their communities and there is no tourism*” (personal communication, October 20, 2020). However, when the poor abandon rural life and move to urban areas, as they have done for decades in Central America, they may live in dense settlements and be forced to venture out daily to earn a miserable livelihood in the “informal sector,” which usually entails selling something in the street [13]. This kind of poverty also entails exposure to an infectious virus.

There are other possible hypotheses to explain national differences in COVID-19 infections and deaths. Perhaps, for example, there are explanations hidden in the “demographic structure” of the populations of each country. However, median ages are similar in the six countries, the population pyramid is not so different, and family structure and living arrangements do not vary significantly. It could be useful to have information about the average level of contact among individuals in each country, but here too it is hard to believe there would be significant national variations. In searching for explanations for the worldwide divergence in COVID-infections and deaths, researchers have wondered if certain previously circulating pathogens can induce a helpful level of immunity, then the specific geography of their reach could explain disparities in the current pandemic. However, the Central American isthmus is both relatively small and has always witnessed considerable trade and movement of people among countries. There are no compelling hypotheses that cry out for testing, and, in any case, the reach and reliability of data are sorely limited. The Central American isthmus adds to the worldwide epidemiological mystery of COVID-19.

## CONCLUSION

An alarming turn-of-events is of political leaders abandoning their responsibility to lead efforts to control the pandemic. Increasingly in the region, governments are shifting their responsibility to citizens taking personal responsibility to tackle COVID-19. In Guatemala, President Alejandro Giammattei gave a speech on August 6, 2020, in which he said, “We have been busy all this time with the coronavirus. I thank God I am leaving this issue now to be able to return to the issue of governing the country. Today we are transferring responsibility to the people. If the people want to take care of themselves, they will take care” [14]. Other countries in the region have reduced efforts at confinement, allowing a resumption of economic and even social activities. The motivation seems driven by political calculations. Weary health care professionals are nervous, not believing that the pandemic has been contained, and certain it has not yet been understood.

The novel coronavirus pandemic has wreaked havoc on a region that has long confronted misfortune. Vaccines are now being offered, and there is hope that suffering will ease. However, a legacy of COVID-19 should be a strengthening of health care systems, including their capacity to collect detailed and uncompromised data.
